# The Influence of Victim Vulnerability and Gender on Police Officers’ Assessment of Intimate Partner Violence Risk

**DOI:** 10.1007/s10896-016-9905-0

**Published:** 2016-12-24

**Authors:** Jennifer E. Storey, Susanne Strand

**Affiliations:** 1 School of Law, Royal Holloway University of London, Egham, Surrey TW20 0EX UK; 2 School of Law, Psychology and Social Work, Örebro University, Örebro, Sweden; 3 Centre for Forensic Behavioural Science at Swinburne University of Technology, Melbourne, Australia

**Keywords:** Victim vulnerability, Male victims, Intimate partner violence, B-SAFER, Violence risk assessment and management, Police

## Abstract

This study investigated the influence of victim vulnerability factors and gender on risk assessment for intimate partner violence (IPV). 867 cases of male and female perpetrated IPV investigated by Swedish police officers using the *Brief Spousal Assault Form for the Evaluation of Risk* (B-SAFER) were examined. For male-to-female IPV, victim vulnerability factors were associated with summary risk judgments and risk management recommendations. For female-to-male IPV, vulnerability factors were more often omitted, and consistent associations were not found between vulnerability factors, summary risk judgments, and risk management. Results indicate that B-SAFER victim vulnerability factors can assist in assessing male-to-female IPV risk. Further research is necessary to examine the use of B-SAFER victim vulnerability factors for female-to-male IPV, as results showed victim vulnerability factors to be less relevant to officers’ decision making, particularly their management recommendations. However, several variables external to the B-SAFER, such as the availability of management strategies may account for these findings.

Intimate partner violence (IPV) includes “any behavior within an intimate relationship that causes physical, psychological, or sexual harm to those in the relationship” (Heise and Garcia-Moreno 2002, p. 89). A recent meta-analysis found the average lifetime prevalence of IPV for women globally to be 30% (Devries et al. 2013). Prevalence varied geographically, and is lower (19.3%) for Western Europe, which includes Sweden where the present study took place. A national survey in Sweden found lifetime victimization rates of 25.5% for women and 16.8% for men aged 16–79. Similar gender differences were found across the type of IPV reported, where psychological violence was reported by 23.5% of women and 14.5% of men and physical violence was reported by 15% of women and 8.1% of men, including sexual violence (5.1% for women and 0.5% for men) (National Council for Crime Prevention, NCCP 2014). The consequences of IPV are varied and severe, impacting hundreds of thousands of individuals each year and affecting a victim’s ability to participate in the community, impacting their mental and physical health, and in some cases causing death (Heise and Garcia-Moreno 2002).

A great deal of advocacy and research aimed at preventing IPV exists. One area in which there has been an exponential rise in research is IPV risk assessment (Messing and Thaller 2013). Violence risk assessment is the process of gathering and analyzing information to characterize risk (e.g., its nature, severity, frequency/duration, imminence, and likelihood). Violence risk assessment is typically followed by violence risk management, which is the process of developing interventions to reduce the risk of violence. More than a dozen IPV risk assessment instruments have been developed. Most of those instruments focus exclusively or in large part on risk factors related to the perpetrator of the IPV.

The *Brief Spousal Assault Form for the Evaluation of Risk* (B-SAFER; Kropp et al. 2005, 2010) is a structured professional judgment (SPJ) violence risk assessment tool for IPV. SPJ tools provide evaluators with critical risk factors derived from the scientific and professional literature and guide evaluators in considering those risk factors to reach a decision about violence risk. The B-SAFER was developed to guide professionals, especially police and other criminal justice professionals, in the assessment and management of IPV. Assessment in this context refers to identifying risk factors and making summary risk judgements of low, moderate or high risk for continued IPV. Management refers to the identification of risk management strategies that mitigate the risks identified in the assessment. Originally, the B-SAFER contained 10 risk factors related to the perpetrator of the IPV divided into two sections: Intimate partner violence (items include Violent acts, Violent threats or thoughts, Escalation, Violation of court orders, Violent attitudes) and Psychosocial adjustment (items include General criminality, Intimate relationship problems, Employment problems, Substance use problems, Mental health problems). In 2010 a third section was added, Victim vulnerability, which includes five victim vulnerability factors related to the victim of the IPV. The vulnerability factors represent the fact that certain factors can place victims at greater risk of continued harm because those factors increase the opportunities for violence and/or make the victim less likely to engage in self-protection. Specifically, the victim may display behaviours toward the perpetrator that are inconsistent or ambiguous, such as making contact despite the existence of a court order, or they may form inconsistent attitudes about the perpetrator as a result of experiencing feelings of minimization, denial, and self-blame (item 11, *Inconsistent attitudes or behaviour)*. The victim’s level of fear can also impede his/her ability and motivation to engage in self-protective actions (Item 12, *Extreme fear of perpetrator*). Victims who are unaware of resources (e.g., laws) or who lack the ability (e.g., are geographically isolated) or motivation (e.g., reluctant to call police) to take advantage of them will also be more vulnerable, which increases the risk of continued IPV (Item 13, *Inadequate support or resources*). Victims without adequate physical security in their home, workplace or transportation, or who continue to reside with the perpetrator are also at higher risk for IPV (Item 14, *Unsafe living situation*). Finally, victims experiencing physical health, mental health, substance abuse, employment or legal problems that increase feelings of helplessness or vulnerability, may have more limited ability or motivation to engage in self-protection (Item 15, *Health problems*).

Discussing, quantifying or considering factors related to a victim that may place them at increased risk of IPV is a controversial but important practice. Controversy often stems from the assumption that considering such factors is akin to victim blaming, or that it places the responsibility of stopping the violence on the victim (Bennett Cattaneo and Goodman 2005). Blaming a victim for a crime that has been committed against him/her is never the intention when considering such factors; the goal is always violence prevention. The B-SAFER victim vulnerability items represent dynamic variables that place the victim at greater risk, but that can also be ameliorated through the implementation of management strategies. For instance, a victim may live in a ground floor apartment with low security or she may live with the perpetrator and this would indicate that she has an *Unsafe living situation.* Identifying the presence of this item in no way implies that the victim is at fault for her victimization, however it does alert authorities to the need to provide the victim with either improved security and resources at her residence (e.g., an alarm system) or an alternate place to reside (e.g., a shelter). IPV is different from many other crimes in that the target of future violence is typically known. As such, it would seem prudent to consider the potential future target when assessing violence risk so that protective services can be focused on his/her needs. In fact, Belfrage and Strand (2008) noted that police officers requested victim-related risk factors to assist them in assessing and managing IPV risk.

Despite an abundance of research on IPV, limited research has been conducted on dynamic (or changeable) risk factors related to victims that may place them at greater risk of future harm (Bennett Cattaneo and Goodman 2005). Other researchers suggest, and we agree, that this may be because doing so can be perceived as victim blaming (Kuijpers et al. 2012a). A small amount of research does exist however. Studies indicate that both members of a couple can increase the risk of IPV (Capaldi and Kim 2007; Moffitt et al. 2001; Sonis and Langer 2008). The vulnerability factors identified for female victims of IPV include: a lack of personal resources, violence perpetrated by the victim against her partner, psychological difficulties, avoidant attachment styles, and a decision by the victim not to engage in the legal process or implement management strategies recommended by police (Hirschel and Hutchinson 2003; Kuijpers et al. 2012a; Kuijpers et al. 2012b; Miller and Krull 1997; Wofford et al. 1994). Protective factors identified include the use of legal or advocacy services and quality of life (Bell and Goodman 2001; Bybee and Sullivan 2002). Based on their findings, both Bennett Cattaneo and Goodman (2005) and Kuijpers et al. (2012b) suggest that victim-related risk factors be considered when conducting IPV risk assessment as a way to prevent future violence.

In addition to limited research on victim-related risk factors, only one study has examined the validity of the B-SAFER victim vulnerability factors. Belfrage and Strand (2008) examined police officer’s use of the B-SAFER in cases of male-to-female IPV. Results indicated that although victim vulnerability factors were less likely to be coded as present than perpetrator risk factors, they were equally related to the summary risk judgments made. In the SPJ approach, risk factors identified as present should be related to summary risk judgments, and both should be related to the risk management strategies implemented. The association between victim vulnerability factors and risk management strategies was not investigated in the study. The authors concluded that victim vulnerability factors have an important role and should be considered when assessing risk for IPV. Although preliminary results support the presence of the victim vulnerability factors in the B-SAFER, additional testing is required to identify whether victim vulnerability factors function equally across genders and whether victim vulnerability factors are related to risk management strategies, these issues will be the focus of the present study.

Within the limited research on victim vulnerability there has been no research to our knowledge on victim vulnerability and gender. Thus, the second focus of the present study is the impact of victim gender on the consideration of victim vulnerability in IPV risk assessment. The existence and nature of IPV perpetrated by females against their male intimate partners is an area of longstanding debate (for a discussion, see Straus 2009). Although evidence indicates that males are much more likely to be arrested as perpetrators of IPV and cause more severe injury, female perpetrators comprise at least a small proportion of all IPV arrests, and in some countries a proportion that has increased in recent years (Henning and Renauer 2005; Straus 2009). Currently, no IPV risk assessment instruments have been developed specifically for female-to-male IPV. As such, practitioners must decide whether to employ instruments designed primarily for male perpetrated IPV or to use unstructured professional judgment (i.e., judgment based on experience and/or qualifications) when assessing IPV risk. This is, of course, problematic given the inaccuracy of unstructured professional judgment and research literature showing gender differences in offender behaviour and criminal justice system outcomes (Henning and Renauer 2005; Monahan 1981).

Recent research has examined the use of the B-SAFER in assessing and managing female-to-male IPV. The B-SAFER can be employed in cases of female-to-male IPV, however, it was developed primarily using the empirical literature on male-to-female IPV, given the paucity of research on the former. Storey and Stand (2013) found that female perpetrators of IPV possessed fewer perpetrator risk factors than male perpetrators, but the risk factors were related to summary risk judgments. Summary risk judgments were not, however, associated with risk management recommendations made by police officers for female perpetrators, but were for male perpetrators. The results indicated that the B-SAFER did not function in the same way for female and male perpetrators of IPV. The study did not include the examination of victim vulnerability, thus it is unclear whether similar results would be found when comparing female and male victims of IPV.

## Current Study

The present study will examine the use of the B-SAFER victim vulnerability factors in IPV risk assessment. This will be accomplished by examining police officers’ use of the B-SAFER to: (a) identify vulnerability factors; (b) make summary risk judgments; and (c) recommend risk management strategies. Further, we will examine whether the gender of the victim impacts these three stages of violence risk assessment and management.

## Method

### Overview

This study employs a true prospective design. Data were gathered in Sweden from three police districts. One district included an urban area in Stockholm which has a population of around 790,000, the two other districts were in central Sweden, one being rural with a population around 250,000 and one remote area with a population around 120,000. Police officers in Sweden are required to conduct violence risk assessments using the B-SAFER in cases of IPV. Police officers receive training in the use of the B-SAFER and in the development of management plans which are lists of recommended risk management strategies for the case. Officers respond to IPV calls, complete their investigation, and then complete the B-SAFER and recommend management strategies for the case. Multiple officers may contribute to the completion of the B-SAFER and management plan. Prior to filing this information in records, a victims of crime officer will review whether the victim requires support, and high priority cases will be taken over by police officers who specialize in security and protection. A victims of crime officer is a police officer or crime analyst who works only with victims who share a personal relationship with the perpetrator. High priority cases are those that require immediate action. For research purposes, the B-SAFER risk assessment and management plans filed and demographic information were retrieved and coded.

### Cases

Between February 27, 2009 and September 17, 2012, 867 IPV cases were reported to police and received a B-SAFER assessment. Victim and perpetrator gender were unevenly distributed within the sample and the implications of this are considered in the discussion. A total of 42 cases involved female-to-male IPV and 825 cases involved male-to-female IPV. For the sake of brevity, we will refer to the individuals investigated for complaints related to IPV as *perpetrators* and the individuals who were their current or former intimate partners as *victims*.

### Procedure

#### Risk Assessment

The B-SAFER was developed to assess and manage IPV risk. The B-SAFER was designed for adult perpetrators of IPV of any gender based on a systematic review of the empirical, clinical, and legal literature. Eight studies have examined the B-SAFER’s validity in samples of male IPV perpetrators. Results indicate that ratings can be made with good interrater reliability and concurrent validity (Au et al. 2008; Belfrage and Strand 2008; de Reuter et al. 2008; Kropp 2008; Kropp and Belfrage 2004; Soeiro and Almeida 2010; Storey et al. 2014; Winkel 2008). The predictive validity of the B-SAFER has been examined in three studies and has shown AUC values around.70 and significant associations between B-SAFER total scores and subsequent psychological and physical violence (de Reuter et al. 2008; Soeiro and Almeida 2010; Storey et al. 2014).

B-SAFER risk and vulnerability factors are coded on a 3-point scale, *No/Absent*, *Possibly/Partially present*, and *Yes/Present*. To facilitate comparisons, researchers converted B-SAFER victim vulnerability factor ratings into numerical scores (*Omit* or *No/Absent* = 0, *Possibly/Partially present* = 1, and *Yes/Present* = 2). Two ratings are made for each perpetrator item, one for the past four weeks (Current) and one for any time prior to that (Past). In Sweden, only Current ratings are made for victim vulnerability factors. Where no valid information is available items may be omitted. Once coded, the evaluator will consider the risk and vulnerability factors and make a summary judgment regarding the risk involved in the case. Two summary risk judgments were made: risk for life-threatening IPV and risk for imminent IPV. Summary judgments are made on a 3-point scale, *Low*, *Moderate,* and *High risk*. Again, to facilitate comparisons, researchers converted summary risk judgments into numerical scores (*Low risk* = 0, *Moderate risk* = 1, *High risk* = 2).

All investigating officers in the cases examined were trained to use the B-SAFER. Training included at least a one-day workshop with theory and case examples, and discussions of both male-to-female and female-to-male IPV. For the cases used in the present study, officers made B-SAFER ratings as part of their work on an active case. As such, it was not feasible to obtain second independent ratings in order to evaluate interrater reliability.

#### Risk Management

Upon completing the B-SAFER, officers documented recommended risk management strategies in their risk management plan. Officers noted that most of the management strategies recommended in their plans were implemented in the case, however based on the file information available we were unable to confirm their implementation. Risk management strategies were coded at the time of the assessment; officers may have recommended additional strategies at a later date. In Sweden, three management strategies are mandatory in all IPV cases: searching the police registry to determine if the perpetrator has prior convictions or access to weapons, and notifying social services if children are present in the home. The most commonly recommended non-mandatory management strategies, and those coded were: initiating a restraining order, protective living (e.g., moving the victim to an apartment with a secret location for a short period of time), a victim support lawyer, and contact with a shelter.

#### Data Analyses

Analyses were conducted using SPSS (Version 22). A series of statistical tests were used depending on the nature of the variables being examined, the analyses included correlation, chi-square, t-test, and ANOVA.

### Demographic Characteristics

Due to the focus on victim vulnerability in the present paper findings are presented based on the gender of the victim. Female perpetrators were 39 years old on average (*SD* = 12.60, range: 19–72), and male perpetrators were 40 (*SD* = 11.96, range: 18–77), this difference was not significant *t*(861) = .36, *p* = .723; information was missing for two females (5%) and five males (<1%). The most serious index offence committed against the majority of male (73%, *n* = 27) and female victims (58%, *n* = 459) was assault, followed by violation of a person’s or woman’s integrity (11%, *n* = 4; 21%, *n* = 167), and unlawful threats (11%, *n* = 4; 15%, *n* = 119). The remaining offences including attempted murder/manslaughter, sexual offenses, and breaking and entering, occurred in a minority of cases (between 3% and <1%). Violation of a person’s integrity is a charge unique to Sweden, it is used when the perpetrator has committed several offences against the victim and is applied as a single more serious overarching charge. On average, males were the victim of 2 (*SD* = .95, range: 1–4) criminal offenses, information was missing in 45% (*n* = 19) of cases. Females were also the victim of 2 (*SD* = 1.05, range: 1–11) criminal offenses on average, information was missing in 40% (*n* = 331) of cases. The mean number of criminal offenses committed did not differ significantly by gender, *t*(515) = .30, *p* = .77.

Being Swedish or of Swedish heritage was defined as either being born in Sweden or having both parents born in Sweden. In the sample of male victims, 52% (*n* = 13) of cases included a victim, perpetrator or both a victim and perpetrator who were not of Swedish decent; information was missing in 41% (*n* = 17) of cases. In the sample of female victims, 56% (*n* = 308) of cases included victims and/or perpetrators who were not of Swedish decent; information was missing in 35% (*n* = 278) of cases. To determine if a statistical difference existed between genders, heritage was dichotomized into any non-Swedish heritage and Swedish heritage. There was no significant difference in heritage based on gender, χ^2^ (1, *N* = 572) = .18, *p* = .671.

At the time that the index offense was reported to police none of the male victims had a pre-existing restraining order against the perpetrator; information was missing in 12% (*n* = 5) of cases. A minority (4%, *n* = 35) of female victims had a preexisting restraining order in place; information was missing in 3% (*n* = 26) of cases.

## Results

### Risk Assessment

#### Individual Victim Vulnerability Factors

The presence of the B-SAFER victim vulnerability factors by gender is displayed in Table 1. The vulnerability factor that was most often endorsed as *Present* for both genders was Item 11 (*Inconsistent attitudes or behaviour*) and the vulnerability factor most often endorsed as *Absent* for both genders was Item 13 (*Inadequate support or resources*). To examine whether significant differences in frequency existed between genders, a Chi-square analysis was conducted. To facilitate this comparison, the presence of items was dichotomized, ratings of *Present* and *Possibly or partially present* were collapsed and compared to *Absent* ratings. Results, presented in Table 1, show no statistically significant differences. Table 1 also compares the frequency of item omission based on gender. Only items 12 (*Extreme fear of perpetrator*) and 13 (*Inadequate support or resources*) showed significant differences. Item 12 was omitted for male victim 2.43 times more often than for female victims, and item 13 was omitted 2.55 more times for male than for female victims.

**Table 1:** Presence and comparison of B-SAFER victim vulnerability factors by gender

B-SAFER Victim vulnerability factors	% (*n*)								χ^2^	
	Male victims (*N* = 42)				Female victims (*N* = 825)				Presence^a^	Omit
	Omit	N	P	Y	Omit	N	P	Y		
11. Inconsistent attitudes or behaviour	26% (11)	24% (10)	21% (9)	29% (12)	17% (139)	34% (278)	18% (151)	31% (257)	.84	2.44
12. Extreme fear of perpetrator	33% (14)	36% (15)	21% (9)	10% (4)	17% (141)	40% (330)	22% (185)	21% (169)	.31	7.18**
13. Inadequate support or resources	45% (19)	41% (17)	10% (4)	5% (2)	25% (202)	45% (367)	14% (115)	17% (141)	2.07	9.06**
14. Unsafe living situation	33% (14)	26% (11)	24% (10)	17% (7)	24% (199)	27% (226)	22% (178)	27% (222)	.12	1.83
15. Health problems	26% (11)	31% (13)	19% (8)	24% (10)	37% (307)	30% (243)	13% (106)	21% (169)	.29	2.09

*N* Absent, *P* Possibly or partially present, *Y* Present. Chi-Square analysis *df* = 1

^a^Ratings of *Present* and *Possibly or partially present* were collapsed and compared to *Absent* ratings

*p ≤ .05, **p ≤ .01, ***p ≤ .001

#### Victim Vulnerability Factor Total Scores

The average victim vulnerability total score for males was 2.97 (*SD* = 2.34, range: 0–10) out of a possible 10, and for females it was 3.41 (*SD* = 2.62, range: 0–10); information was missing in 12% (*n* = 5) and 1% (*n* = 11) of cases respectively. The mean values did not differ significantly based on gender, *t*(813) = .99, *p* = .32. The mean number of victim vulnerability items omitted also did not differ significantly between male (*M* = 1.64, *SD* = 1.77) and female victims (*M* = 1.20, *SD* = 1.54), *t*(865) = −1.81, *p* = .070). Internal consistency of the victim vulnerability scores was measured using coefficient alpha. Coefficient alpha for the victim vulnerability factors among male victims was .78 and for female victims was.72 which is considered acceptable according to George and Mallery (2003).

#### Summary Risk Judgments

Table 2 presents associations between the mean number of victim vulnerability factors identified in a case and summary risk judgments of imminent and life-threatening risk. For male victims, the association between vulnerability total scores and imminent risk ratings was significant and large in size (Cohen 1988). Post hoc tests revealed that low risk cases had significantly fewer vulnerability factors than both moderate and high risk cases. For female victims, the associations between vulnerability total scores and summary risk judgments were significant and medium in size. Post hoc tests for life-threatening risk revealed that low risk cases had significantly fewer vulnerability factors than moderate or high risk cases. Post hoc tests for imminent risk revealed significant differences in mean scores across all three levels of risk in the expected direction, where high risk cases had significantly more vulnerability factors than moderate and low risk cases, and moderate risk cases had significantly more vulnerability factors than low risk cases.

**Table 2:** Association between victim vulnerability total scores and summary risk judgments

Victim gender	Summary risk judgments	Total score *M(SD)*			ANOVA		
		Low	Moderate	High	*df*	*F*	η^2^
Male	Life-threatening	2.44 (2.10)	4.13^a^ (2.80)	4.67^a^ (2.08)	(2, 35)	2.56	.13
	Imminent	1.76 (1.44)	3.73 (2.41)	4.63^a^ (2.72)	(2, 35)	6.09**	.27
Female	Life-threatening	2.73 (2.33)	4.59 (2.63)	5.22 (2.64)	(2, 764)	59.18***	.13
	Imminent	2.22 (2.19)	3.68 (2.41)	5.19 (2.57)	(2, 765)	87.93***	.19

^a^ *n* < 10

*p ≤ .05, **p ≤ .01, ***p ≤ .001

### Risk Management

In cases with male victims, police recommended at the time of assessment that a restraining order be initiated in 17% (*n* = 7) of cases, a victim support lawyer was recommended in 17% (*n* = 7) of cases, a shelter in 7% (*n* = 3), and protective living was recommended in 5% (*n* = 2) of cases. A mean of one strategy (*SD* = .92, range: 0–3) was recommended per case, and no strategies were recommended in 45% (*n* = 19) of cases. For female victims, police recommended the initiation of a restraining order in 23% (*n* = 187) of cases, a victim support lawyer in 19% (*n* = 160) of cases, a shelter in 11% (*n* = 98), and protective living was recommended in 10% (*n* = 80) of cases. An average of one strategy was recommended per case (*SD* = .92, range: 0–4), no recommendations were made in 41% (*n* = 339) of cases. No significant difference existed between the number of strategies recommended by gender, *t*(711) = .93, *p* = .355.

Mean total scores for victim vulnerability factors were compared to risk management recommendations (see Table 3). No significant correlations were found between victim vulnerability scores and risk management for male victims. By contrast, two risk management strategies as well as the total number of strategies recommended were positively and significantly associated with the number of victim vulnerability factors in the case.

**Table 3:** Correlation between victim vulnerability total scores and recommended risk management strategies at the time of assessment

Victim gender	Risk management strategies				Total number of risk management strategies
	Restraining order	Protective living	Victim support lawyer	Shelter	
Male	.01	.16	.23	.42	.26
Female	.06	.21***	.19***	.09	.19***

*p ≤ .05, **p ≤ .01, ***p ≤ .001

Summary risk ratings were compared to the total number of recommended management strategies. For male victims, only the risk of life-threatening violence was significantly associated with the number of risk management strategies recommended, *r* = .37, *p* = .044. In contrast, for female victims the number of management strategies recommended was significantly associated with ratings of both life threatening risk, *r* = .32, *p* < .001, and imminent risk, *r* = .27, *p* < .001, demonstrating that as risk increased the number of management strategies recommended also increased.

## Discussion

Results revealed positive findings related to officers’ use of victim vulnerability factors in their work on IPV cases. Victim vulnerability factors were more often omitted in cases with male victims, but otherwise showed no difference in presence across gender. In all but one instance, officers’ determinations of overall risk were positively related to the number of victim vulnerability factors present, as would be expected in the SPJ approach. The number of risk management strategies recommended did not differ by gender. In cases with female victims, officers’ risk management recommendations were related to the presence of victim vulnerability factors and level of overall risk identified, again in keeping with the SPJ approach. In cases with male victims, officers’ risk management recommendations were not consistently related to victim vulnerability or risk level. Although gender differences were identified in how the B-SAFER was used, these differences may be unrelated to the B-SAFER and more aptly explained by other variables such as the availability of risk management strategies for male victims of IPV and low power due to the small sample size.

The presence of victim vulnerability factors did not differ by gender; however, the number of omitted items did. In both instances where differences in item omission were identified, police were more than twice as likely to omit vulnerability factors for male victims. The reason for the omissions is unclear, two possibilities seem plausible given the two items that were more often omitted. First, police may have omitted the item because they were unable to obtain evidence regarding the item. For instance, males may have been reluctant to discuss feeling fearful, making Item 12 (*Extreme fear of perpetrator*) difficult to code. Alternatively, police might have failed to understand how a male victim could be extremely fearful of a female perpetrator. Second, police may have felt unable to code the item. For instance, police might have been unsure of how to code Item 13 (*Inadequate support or resources*) because they might have thought that the resources typically recommended for female victims were unavailable or unsuitable for male victims. It is important to ascertain the reasons for the omissions because at the moment they could be interpreted as problems with the scoring of the B-SAFER, but most likely reflect social problems, such as stereotypes of masculinity and resource availability. Internal consistency for victim vulnerability factors for both male and female victims were in the acceptable range suggesting that items were measuring a single construct, IPV risk.

Victim vulnerability total scores were in all but one instance related to overall risk ratings. These results indicate that officers were considering victim vulnerability when making summary risk judgments as is proper procedure when using the B-SAFER. Victim vulnerability total scores for male victims were not significantly related to judgments about life-threatening risk. There are several possible explanations for this finding. The first, and most likely explanation, is that the sample size for this analysis was too small to detect an effect. Despite not reaching significance, the results followed the expected pattern, with lower mean total scores for low risk ratings and higher scores for high risk ratings, suggesting that with a larger sample size the analysis might have reached significance. Second, because life-threatening violence is a relatively rare outcome and officers would not have been as experienced using the B-SAFER with male victims (given the low prevalence of male victims in the present sample) they were unable to use the B-SAFER in a consistent manner when assessing risk for life-threatening violence. Third, officers may have been considering vulnerability factors not included in the B-SAFER when evaluating the risk for lethal violence toward male victims, and as such these factors would not have been included in total scores.

When risk management was examined it was found that, unlike females, no male victims had restraining orders in place prior to the index offense. Yet, no difference existed in the total number of risk management strategies recommended by gender. Victim vulnerability total scores were not related to risk management for male victims. This lack of association is of concern because several of the risk management strategies examined are directly related to victim vulnerability (e.g., protective living and Item 14 *Unsafe living situation*). As such, we would hope that police consider these vulnerability factors when recommending risk management strategies. The lack of association is also of concern because in the SPJ approach risk and vulnerability factors indicate areas where management is needed, in other words, management is used to mitigate the risk identified. The results of Table 3 shed further light on these findings because they also show a gender difference where summary risk judgments are consistently associated with risk management for female victims but not for male victims. Thus, victim vulnerability factors for females functioned as expected in the SPJ approach. Specifically, as the presence of vulnerability factors increased, so too did judgments of overall risk and the amount of management recommended.

There are several possible explanations for why vulnerability total scores for male victims were not related to risk management recommendations. First, the limited significant findings may reflect low power due to sample size. Second, the vulnerability factors are not viewed by police as being as applicable to males as they are to females, and thus are not treated as important targets for management. Third, police may have felt that the vulnerabilities possessed by the male victims were not as amenable to the management strategies available, since those strategies were originally developed for female victims of IPV. Fourth, some of the male victims may have also been perpetrators of IPV. If the male victims had also engaged in IPV, police may not have taken their claims of victimization seriously and therefore may not have recommended a level of management equivalent to the risk identified.

This study has some limitations that should be considered when interpreting the findings. The first, is the small number of male victims. The implications of this limitation are discussed above. We wish to add that the most likely implication of this limitation is that significant effects were not detected (Type II error), meaning the results may provide an overly negative portrayal of the B-SAFER and management recommendations when used for male victims. Efforts were made to obtain a larger sample including a lengthy data collection period of 3.5 years, and the collection of data from three jurisdictions to ensure that police practices did not influence the number of cases where a B-SAFER was used. Future studies may need to devise different methods to obtain larger samples.

The second limitation is that the sample consisted of cases reported to police which, as Devries et al. (2013, p. 1527) note, is not the “gold standard” research method for IPV. The sample is therefore most probably an underestimate of the number of instances of IPV that occurred in the three jurisdictions during the time period examined, and may not be representative of all IPV. Specifically, we would anticipate that victims who did not report abuse to police would have more victim vulnerability factors, particularly given that not reporting IPV is considered under Item 11 and to some extent also under Item 13. Nevertheless, the study design was advantageous because it assessed the use of the B-SAFER within actual police practice. Third, we were unable to confirm whether recommended management strategies were implemented, officers could only state that the strategies they recommend are typically implemented. Finally, we were unable to determine inter-rater reliability for the B-SAFER. Although not ideal, these types of limitations are often unavoidable when conducting field research. Further, despite these limitations, the present study provides unique findings on an understudied area that can be used to guide future research.

### Implications for Practice

The present study has both specific and general implications for practice. Generally, the results provide support for the consideration of victim vulnerability in the assessment and management of IPV. Although different violence risk assessment instruments will assess victim vulnerability in different ways, the present results reveal that this can be done in practice by police officers and that the vulnerabilities identified are being linked to risk and management decisions. This suggests that such assessments are assisting officers in their duties and resulting in a distribution of resources that reflect victim needs.

More specifically, the present results shed light on an important question related to the use of the B-SAFER in practice. Can victim vulnerability be considered in IPV risk assessment equally across genders? Our results reveal, for the first time, an association between victim vulnerability factors, summary risk judgments and risk management recommendations. Further, they show that officers are able to include these factors in a structured consideration of risk and management in cases with female victims. Additional research is required to determine the predictive validity of the victim vulnerability factors but the current results support the utility of the factors.

In accordance with Storey and Stand (2013), the B-SAFER did not function equally across genders. The results indicate some problems with coding vulnerability factors, and that vulnerability factors were less relevant to police decision making. Although, it is notable that the former coupled with low sample size may have been the cause of the latter, additional research is required to draw firm conclusions. Until such research is available, the authors suggest that individuals providing training on the B-SAFER take time to speak to trainees about male victims with special attention paid to items 12 and 13 and the process of linking victim vulnerability factors, summary risk judgments, and risk management.

Limited associations were found between risk assessment and management, meaning that for female-to-male IPV risk factors for both victims and perpetrators were generally unrelated to risk management recommendations. This is concerning because the Risk Needs Responsivity or RNR model holds that improved correctional outcomes are the result of pairing the level of risk to the needs and abilities of the perpetrator (Andrews and Bonta 2006; Andrews et al. 2006). Thus, we would ideally want to see that the risk factors and risk level present in a case are paired with the level of risk management recommended. The results herein suggest that the greater problem lies with risk management for female-to-male IPV. As such, we suggest that more practical attention be paid to the identification and/or development of risk management strategies for male victims of IPV, as well as training for police around recommending management for this group. Prior research has shown that resources are less available for male victims of IPV, and that in some instances police are less likely to take action in cases of female-to-male IPV (Douglas and Hines 2011; Drijber et al. 2013). Further, Douglas and Hines (2011) found that more than half of male victims who sought help from police found them to be “not at all helpful”. This finding by Douglas and Hines (2011) may be somewhat explained by the mismatch between risk and management found in the present results, where victims may view risk management strategies that do not correspond to risk factors or risk level as unhelpful. This problem is an important one for practice as poor or limited management will lead to greater rates of IPV recidivism and heightened rates of post-traumatic stress disorder among victims (Belfrage et al. 2011; Douglas and Hines 2011).

### Implications for Research

The results related to victim vulnerability factors when used with female victims were very promising. Future research should continue this work by examining the predictive validity of the B-SAFER victim vulnerability factors. In addition to validating the B-SAFER and the victim vulnerability factors, this type of research could highlight particular victim vulnerabilities that should be the focus of risk management efforts.

With respect to male victims, the results indicate that victim vulnerability ratings were not always consistently related to overall risk ratings and were unrelated to risk management. These findings raise questions that require follow-up data on IPV recidivism and additional information on male victims to answer. Such information could reveal several things. First, recidivism data may reveal that some male victims are also perpetrators of IPV and that many of the female perpetrators come to police attention again as victims. In fact, Henning et al. (2009) found that female perpetrators were five times as likely to come to police attention again as victims of IPV, whereas male perpetrators tended to return as perpetrators. This finding provides support to the prior suggestion that officers may have known some of the male victims to also be perpetrators of IPV, and thus might not have seriously considered their vulnerabilities or recommended the types of risk management strategies examined herein. Second, additional information on the circumstances of the male victims or the nature of the resources available to them might reveal that the risk management strategies investigated are less suited for male victims, making it difficult for police to pair high risk with high management. For instance, police may have failed to recommend certain strategies because they knew the purveyors of the resource in question would not assist men. For instance, male victims in a study by Douglas and Hines (2011) reported being ridiculed, accused of being a batterer, and feeling that they were not supported. General research on male victims and resources for male victims are sorely needed, and research that investigates recidivism while also gathering information on the characteristics of the male victims and resources will shed more light on the use of the B-SAFER with this population and the efficacy of risk management strategies.

## Conclusion

The results suggest that the B-SAFER victim vulnerability factors can be used in practice as intended with female victims of IPV. Specifically, vulnerability factors were related to summary risk judgments, which were related to risk management recommendations. Further, the data were derived from actual police practice, indicating that police officers have the ability and information required to code these vulnerability factors and utilize them to assess and manage risk in active IPV cases. The same results were not found for male victims of IPV, where it appeared that the B-SAFER victim vulnerability factors were less relevant to officers’ decision making. Further research is required to fully understand the role of victim vulnerability factors in assessing and managing IPV risk for male victims with particular emphasis on how officers are identifying appropriate risk management for male victims.
